# Unveiling the *Launaea nudicaulis* (L.) Hook medicinal bioactivities: phytochemical analysis, antibacterial, antibiofilm, and anticancer activities

**DOI:** 10.3389/fmicb.2024.1454623

**Published:** 2024-10-03

**Authors:** Fathy M. Elkady, Bahaa M. Badr, Amr H. Hashem, Mohammed S. Abdulrahman, Amer M. Abdelaziz, Abdulaziz A. Al-Askar, Gehad AbdElgayed, Hany R. Hashem

**Affiliations:** ^1^Department of Microbiology and Immunology, Faculty of Pharmacy (Boys), Al-Azhar University, Cairo, Egypt; ^2^Department of Basic Medical and Dental Sciences, Faculty of Dentistry, Zarqa University, Zarqa, Jordan; ^3^Department of Medical Microbiology and Immunology, Faculty of Medicine, Al-Azhar University (Assiut Branch), Assiut, Egypt; ^4^Department Botany and Microbiology, Faculty of Science, Al-Azhar University, Cairo, Egypt; ^5^Department of Botany and Microbiology, Faculty of Science, King Saud University, Riyadh, Saudi Arabia; ^6^Integrated Molecular Plant Physiology Research, Department of Biology, University of Antwerp, Antwerp, Belgium; ^7^Department of Microbiology and Immunology, Faculty of Pharmacy, Fayoum University, Al-Fayoum, Egypt

**Keywords:** *Launaea nudicaulis*, plant extract, *P. aeruginosa*, MIC, MBC, antibiofilm activity, anticancer activity

## Abstract

Commonly used antimicrobial agents are no longer effective due to their overuse or misuse. In addition, many medicinal plant extracts can combat infectious diseases due to their main active constituents or secondary metabolites. The current study aimed to assess the bioactivities of *Launaea nudicaulis* (LN) leaf extract (LE) against different multi-drug resistant (MDR) *Pseudomonas aeruginosa* (*P. aeruginosa*) isolates. The ethyl acetate extract of a *Launaea nudicaulis* (LN) leaf was analyzed using GC–MS, which identified 27 key bioactive compounds. The major constituents found were as follows: 7-acetyl-6-ethyl-1,1,4,4-tetramethyltetralin, isopropyl myristate, thiocarbamic acid, N,N-dimethyl, S-1,3-diphenyl-2-butenyl ester, hahnfett, cyclopentane acetic acid, 3-oxo-2-pentyl-, methyl ester, hexadecanoic acid, and dotriacontane. Our study demonstrated that the LN leaf was a rich source of other important phytochemicals, including phenolic acids, tannins, saponins, and steroids. The relative biosafety of the *L. nudicaulis* LE was determined from the elevated inhibitory concentration 50 (IC50) of 262 μg/mL, as calculated from the cytotoxicity assay against the Wi-38 normal cell line. Conversely, 12.7 and 24.5 μg/mL were the recorded low IC50 values for the tested extract against the MCF-7 and Hep-G2 cancerous cell lines, respectively, reflecting its potent activity against the tested cancerous cell lines. Microbiologically, the susceptible *P. aeruginosa* isolates to the tested extract showed a growth inhibition zone diameter, in the well diffusion assay, ranging from 11.34 ± 0.47 to 26.67 ± 0.47 mm, and a percent inhibition (PI) value of 50–106.2%, reflecting its acceptable activity. In addition, the broth microdilution assay recorded minimum inhibitory concentration (MIC) and minimum bactericidal concentration (MBC) in the ranges of 15.625–1,000 μg/mL and 125–1,000 μg/mL, respectively. In conclusion, the *L. nudicaulis* LE revealed showed promising activity and high selectivity against *P. aeruginosa*. Moreover, the extract exhibited natural anticancer activities with safe low concentrations, indicating its potential as a superior candidate for future studies of its active constituents.

## 1 Introduction

The overuse or misuse of antimicrobial agents in different sectors, particularly clinical healthcare, animal treatment, and agricultural practices, has resulted in the emergence of antimicrobial resistance. As a result, commonly used anti-infectious agents are no longer effective, posing a serious global health concern in the 21st century (Ahmed et al., 2024). *Pseudomonas aeruginosa* is a highly adaptable bacterium, with a potential risk of developing increased resistance to currently used antimicrobials. Consequently, this pathogen is among the top pathogens that develop a multi-drug resistant (MDR) phenotype in response to antimicrobial misuse or overuse (Coșeriu et al., 2023). Hence, the Infectious Diseases Society of America (IDSA) categorized *P. aeruginosa* as a causative agent of difficult-to-treat infections (Sathe et al., 2023). Although g/mL were classified common carbapenems are used as a last line of defense to control severe infections, the Centers for Disease Control (CDC) has announced the urgent need to monitor bacterial carbapenem resistance (Coșeriu et al., 2023). The WHO included carbapenem-resistant *P. aeruginosa* on its pathogens list, emphasizing the critical need for innovative therapeutic strategies against the listed pathogens (El-Sapagh et al., 2023).

Plant extracts have long been recognized as a source of antimicrobial compounds, with many species producing secondary metabolites such as phenolics, terpenoids, alkaloids, and flavonoids that exhibit broad-spectrum activity against bacteria, fungi, viruses, and even protozoa (Cowan, 1999; Vaou et al., 2021; Yetgin, 2024). These plant-derived antimicrobials can disrupt microbial cell membranes, inhibit enzymes, and interfere with other vital cellular processes, making them potentially useful in various applications, including food preservation, healthcare, and agriculture (Khare et al., 2021). The complex and diverse nature of plant-derived antimicrobials can also make it more challenging for microbes to develop resistance as they would need to overcome multiple mechanisms of action simultaneously (Abdallah et al., 2023). In addition, the evolving application of antibacterial phytochemicals against bacterial biofilms resulted from the increasing research on the activity of these natural products (Wu et al., 2024). The use of such products has several benefits, including enhanced patient tolerance, effortless biodegradability, renewability, relatively low cost, minimal side effects, and acceptability (Paneri et al., 2023).

The methanolic extract of *Launaea mucronata* showed remarkable antimicrobial activity against *Candida albicans* and *Proteus vulgaris*. The extract also exhibited high antioxidant and biosafety levels and high activity against hepatocellular cancerous cell lines (Abouzied et al., 2021).

Similarly, *Launaea nudicaulis* (LN) extract showed strong free radical scavenging activity and demonstrated selective antiproliferative effects against different cancer cell lines compared to normal cells. In addition, the LN methanolic extract demonstrated significant natural insecticide effects (Abouzied et al., 2021).

This study aimed to identify the key compounds in *L. nudicaulis* leaf extract (LE) using GC–MS and phytochemical analyses and aimed to examine its antibacterial and antibiofilm activities against various MDR *P. aeruginosa* clinical isolates. In addition, the study aimed to evaluate the extract’s cytotoxic activities toward both normal and cancerous cell lines.

## 2 Materials and methods

### 2.1 Identification of the clinical isolates

Primarily identified 24 MDR *P. aeruginosa* clinical isolates from patients with infected wounds were obtained from the microbiology laboratory at Sayed Galal University Hospital, Al–Azhar University, Cairo, Egypt. The *Pseudomonas aeruginosa* isolates were identified according to the conventional standard guidelines described by Procop et al. (2017). These standards include initial culturing of clinical specimens on nutrient agar, 5% blood agar, and MacConkey agar, followed by aerobic incubation for 24 h at 37°C. Subsequently, the colony morphology, motility, Gram-stain reaction, and bacterial cell shape and arrangement were examined. Furthermore, the pigment production, the aerobic growth at 40 and 42°C on MacConkey agar, and the growth behavior on cetrimide and TSI agars were evaluated. In addition, the ability of the tested isolates to produce catalase, oxidase, citratase, and tryptophanase enzymes was assessed. Finally, a phenotypic evaluation of the obtained *P. aeruginosa* clinical isolates’ susceptibility to imipenem was aseptically performed using the broth microdilution protocol described by the Clinical and Laboratory Standard Institute (2020) recommendations. The assayed isolates with an imipenem minimum inhibitory concentration (MIC) of ≥8 μg/mL were classified as carbapenem-resistant *P. aeruginosa*.

### 2.2 Plant material and preparation of the crude extract of *L. nudicaulis* leaves

*Launaea nudicaulis* (L.) Hook was collected from the arid region at Ain-Sokhna Road, Suez, Egypt. The identification of the plant used in this study was carried out by Prof. Dr. Abdou Marie Hamed at the Botany and Microbiology Department, Faculty of Science, Al-Azhar University, Cairo, Egypt. All experimental research and field studies on plants, including the collection of plant materials, comply with relevant institutional, national, and international guidelines and legislation.

Dried LN leaves (20 g) were extracted using 100 mL of ethyl acetate as a solvent. The mixture was allowed to stand for 72 h at room temperature, and then, it was filtered using a Whatman No. 2 filter paper. After the filtration stage, the filtrate was evaporated and left to dry at room temperature. Then, the crude extract was stored at 4°C until use (Gennaro, 2008).

### 2.3 GC–MS analysis

The leaf extract of LN was first solubilized in CH_3_OH, then dried using anhydrous Na_2_SO_4_, and finally passed through a 0.45 μm syringe filter before injection into a GC–MS system. The GC–MS system used was a Trace GC Ultra-ISQ system (Thermo Scientific, United States). The initial column temperature was set at 70°C, then increased to 280°C at a rate of 5°C per min and held for 2 min, followed by a further increase to 300°C at a rate of 10°C per min. The extracted components were identified and quantified by comparing their mass spectra and retention times to the databases of known compounds from the Wiley 09 and NIST 11 libraries (Rivero-Cruz et al., 2020).

### 2.4 Phytochemical analysis

The test samples were subjected to phytochemical analysis to determine qualitatively the presence of chemical constituents, as described in the works of Trease and Evans (1989), Harborne (1998), and Sofowara (1993).

#### 2.4.1 Total phenolic acids content determination

The total phenolic content (TPC) was determined using the Folin–Ciocalteu (FC) method, as described by Makkar (2003). Fifty μL of the *L. nudicaulis* LE was aliquoted into test tubes, and the volume in each tube was adjusted to 1 mL using distilled water. To each tube, including the blank one, 0.5 mL of FC (1 N) was added and allowed to stand for 5 min at 25°C. Then, 2.5 mL of a 5% Na_2_CO_3_ solution was added to all the tubes, including the blank one. The samples were incubated in the dark for 40 min at 25°C. The absorbance of the samples was measured at 725 nm using a spectrophotometer.

#### 2.4.2 Total flavonoid content determination

The total flavonoid (TF) content was determined using AlCl_3_, as described by Zhishen et al. (1999). The *L. nudicaulis* LE (0.5 mL) was pipetted into a series of test tubes, and the volume in each tube was adjusted to 1 mL with distilled water. Then, 150 μL of a 5% NaNO_2_ solution was added to all the tubes, and the tubes were allowed to incubate for 5 min at 25°C. Next, 150 μL of a 10% AlCl_3_ solution was added to all the test tubes, and the tubes were incubated for 6 min at 25°C. After this, 2 mL of a 4% NaOH solution was added to all the tubes, and the final volume was made up to 5 mL with distilled water. The test tubes were vortexed and allowed to stand in distilled water for 15 min. The absorbance of the samples was measured at 510 nm using a spectrophotometer.

#### 2.4.3 Total tannins content determination

The tannin content (TC) was determined using the Folin–Denis (FD) method, as described by Makkar (2003). To measure the non-tannin phenolics (NTP), 0.5 mL of the LE and 0.5 mL of distilled water were added to 0.1 g of polyvinyl polypyrrolidone (PVPP) at 0°C. The mixture was then incubated at 4°C for 4 h and centrifuged for 10 min. The supernatant, which contained the NTP, was collected. For tannin determination, 0.5 mL of the FD reagent (1 N) and 100 μL of the NTP extract were combined, and the volume was made up to 1 mL with distilled water for each sample, including the blank. After mixing, the samples were allowed to stand at 25°C for 5 min. Then, 2.5 mL of a 5% Na_2_CO_3_ solution was added to all the test tubes, including the blank. The tubes were mixed again and incubated at 25°C for 40 min in the dark. The absorbance of the samples was measured at 725 nm using a spectrophotometer.

#### 2.4.4 Total flavonol content determination

The total flavonol content in the *L. nudicaulis* LE was determined using AlCl_3_, as described by Miliauskas et al. (2004). To the sample, 2 mL of an AlCl_3_ solution and 6 mL of a sodium acetate (CH_3_COONa) solution were added. The mixture was then incubated at 20°C for 2 h. The absorbance of the samples was measured at 440 nm using a UV–Vis spectrophotometer.

#### 2.4.5 Total steroid content determination

A 5 g sample of the dried *L. nudicaulis* extract was weighed and hydrolyzed by boiling it in 50 mL of a hydrochloric acid solution for 30 min. The solution was then filtered. An equal volume of ethyl acetate was added to the filtrate, and the mixture was mixed well and allowed to separate into layers. The ethyl acetate layer, containing the extracted compounds, was recovered, while the aqueous layer was discarded. The ethyl acetate layer was dried for 5 min at 100°C in a steam bath, and the steroids were then extracted by heating with concentrated amyl alcohol. This resulted in a turbid mixture. The mixture was weighed, and the steroid content was calculated and expressed as mg/100 g of dry weight of the plant sample, as described by Harborne and Harborne (1973).

#### 2.4.6 Total alkaloid content determination

The total alkaloid content of the dried *L. nudicaulis* extract was measured quantitatively using the method described by Harborne and Harborne (1973). A 1 g sample of *L. nudicaulis* powder was mixed with a 4:1 ratio of 70% ethanol and glacial acetic acid. The mixture was allowed to stand for at least 6 h and then filtered. The alkaloids present in the supernatant were precipitated by the dropwise addition of a concentrated ammonia solution. The precipitated alkaloids were then filtered on a pre-weighed scale and dried in an oven at 70°C until they reached a constant weight. The alkaloids content was calculated and expressed as mg/100 g of dry weight of the plant sample.

#### 2.4.7 Total saponin content determination

Approximately 50 mg of the dried *L. nudicaulis* extract was added to 25 mL of 20% ethanol. The sample was heated on a magnetic stirrer for 4 h at a temperature of 55°C, with continuous stirring. The mixture was then filtered, and the residue was re-extracted with an additional 50 mL of 20% ethanol. The filtrates were combined and concentrated at 90°C to a volume of 10 mL. The concentrated filtrate was then mixed with 5 mL of diethyl ether, and the diethyl ether layer was removed. The remaining aqueous layer was stored. This purification step was repeated. The remaining aqueous layer was then mixed with 15 mL of butanol, forming two layers. The butanol layer was separated, and the aqueous layer was filtered again with an additional 15 mL of butanol. The combined butanol layers were mixed with 5 mL of 5% NaCl. The solution was then evaporated in a water bath, and the sample was dried in an oven until a constant weight was reached. The saponin content was calculated and expressed as mg/100 g of dry weight of the plant sample, as described by Ajiboye et al. (2013).

### 2.5 Cytotoxicity and anticancer effects

The growth inhibitory activity of the *L. nudicaulis* LE was evaluated using 3-(4,5-dimethylthiazol-2-yl)-2,5-diphenyletetrazolium bromide (MTT) assay, as described by van de Loosdrecht et al. (1994). Normal human diploid (WI-38) cell lines were used for the assessment of the cytotoxic effect, while breast cancerous (MCF-7) cell lines and hepatocellular carcinoma (Hep-G2) cell lines were used to determine the anticancer activity of the *L. nudicaulis* LE. The optical density (OD) of each tested cell line at 560 nm was measured and used for cell viability or inhibitory percentage calculation, following Equations 1 or 2, respectively.


(1) $$\mathrm{Viability}\;\%=\frac{\mathrm{Test}\;OD}{\mathrm{Control}\;OD}\times 100$$

(2) Inhibition%=100−Viability%

### 2.6 Antibacterial activity of the *L. nudicaulis* LE against the *P. aeruginosa* clinical isolates

For the preparation of the inoculum of each tested *P. aeruginosa* clinical isolate, several pure colonies of a fresh culture were aseptically suspended in sterile normal saline and adjusted to 0.5 McFarland standard turbidity (1 × 10^8^ CFU/mL).

#### 2.6.1 Primary screening for antibacterial activity

The primary *in vitro* activity of the *L. nudicaulis* LE against the MDR *P. aeruginosa* isolates was evaluated using agar well diffusion assay, which was conducted as three independent tests, according to Assefa et al. (2024). In brief, each sterile Mueller–Hinton agar (MHA) plate was aseptically inoculated with each tested bacterial inoculum using a sterile cotton swab to obtain a uniform lawn bacterial growth form. Then, on the agar surface, equidistance three wells were made using a sterile borer of a diameter of 6 mm. One well was filled with 80 μL of the 2000 μg/mL tested *L. nudicaulis* LE dissolved in dimethyl sulfoxide (DMSO), while the wells containing DMSO and the wells containing ceftriaxone (30 μg/mL) were included as negative controls and positive controls, respectively. Before aerobic incubation at 37°C for 24 h, the *L. nudicaulis* LE was allowed to pre-diffuse into the MHA by keeping the inoculated plates in a refrigerator for 30 min. Each inhibition zone diameter (IZD) was measured in millimeters (mm), calculated as the mean of triplicate results, and presented as the mean ± standard deviation (mean ± SD). Finally, the activity index (AI) for the *L. nudicaulis* LE against each tested isolate was calculated using Equation 3. In addition, the percent inhibition (PI) of the *L. nudicaulis* LE against the tested isolates was estimated using Equation 4, as described by Munazir et al. (2012).


(3) $$\mathrm{Activity Index}\;\left(\mathrm{AI}\right)=\frac{IZD\;\mathrm{caused}\;by\;\mathrm{the}\;PE}{IZD\;\mathrm{caused}\;by\;\mathrm{the standard antimicrobial}}$$

(4) $$\mathrm{Percent Inhibition}\;\left(\mathrm{PI}\right)=\mathrm{AI}\times 100$$

#### 2.6.2 Quantitative antibacterial activity

The minimum concentrations of the tested *L. nudicaulis* LE required to inhibit the growth or destroy the tested clinical isolates were inspected using the broth microdilution method (CLSI, 2020). In brief, a stock solution of the tested *L. nudicaulis* LE (1,000 μg/mL) in DMSO was prepared, and a 2-fold serial dilution was conducted in each row of sterile 96-well microtiter plates using sterile Mueller–Hinton broth (MHB). In each row, 10 μL of the tested clinical isolate inoculum was added to each tested well. In addition, wells containing MHB and DMSO inoculated with the tested isolate without the *L. nudicaulis* LE served as positive controls, while the negative control consisted of un-inoculated MHB containing the *L. nudicaulis* LE and DMSO. After incubation at 37°C for 24 h, 40 μL of a resazurin solution (0.015%) was added to each well, and the plates were incubated for 2 h at 37°C. In each row, the lowest concentrations of the *L. nudicaulis* LE that exhibited no observable change in the color of resazurin to red were designated as the MIC. A 10 μL suspension from the wells representing the MIC and the higher concentrations was inoculated onto MHA, followed by incubation at 37°C for 24 h. The lowest concentration causing no visible bacterial growth on the MHA was designated as the minimum bactericidal concentration (MBC) of the *L. nudicaulis* LE. The interpretation of the antibiosis effect of the tested *L. nudicaulis* LE, either bacteriostatic or bactericidal, was clarified after the calculation of the MIC index (MIC_i_) using Equation 5. An MIC_i_ value of ≤2 indicated the extract’s bactericidal activity, while an MIC_i_ value of ≥4 indicated its bacteriostatic effect (Shehabeldine et al., 2023)


(5) $$\mathrm{MICi}=\frac{MBC}{\mathrm{MIC}}$$

In addition, to illustrate the antibacterial activity of the *L. nudicaulis* LE caused either by its selective toxicity toward the tested microorganisms or due to its general toxic effect, the selectivity or therapeutic index (TI) of the *L. nudicaulis* LE was calculated, as described by Adamu et al. (2014) following Equation 6.


(6) $$\mathrm{Therapeutic index}=\frac{\mathrm{The}\;LE\;\mathrm{cytotoxicity}}{\mathrm{MIC}}$$

### 2.7 Antibiofilm assay

The sterile 96-well microtiter plate was used for crystal violet (CV) assay to evaluate the biofilm formation level and the activity of the *L. nudicaulis* LE against various *P. aeruginosa* biofilm formations. In brief, several colonies of each tested bacterial isolate were inoculated into MHB and incubated at 37°C for 24 h. Each bacterial culture of the 0.5 McFarland standard (1.5 × 10^8^ CFU/mL) was then diluted 1:100 using the sterile MHB. For the determination of the biofilm formation category, a triplicate assay for each tested isolate cultured on the MHB was incubated at 37°C for 24 h, where the wells containing bacterial untreated MHB is considered negative control (N). To determine the effect of the tested *L. nudicaulis* LE on the biofilm formation, the same assay was conducted in the presence of a 0.5 MIC of the plant extract. After discarding the planktonic bacterial suspension and the excess nutrient medium, the OD at 620 (OD_620_) of each CV–stained bacterial biofilm was determined using an ELISA reader. The optical density cut-off (ODc) of the negative control was calculated as the mean OD plus three times the SD (Kamali et al., 2020). Bacterial categorization into non- (OD < ODc), weak (OD < OD < 2ODc), moderate (2OD < OD < 4ODc), or strong (OD > 4ODc) biofilm forming was then recorded. In addition, the obtained OD was used to calculate the percentage change in the biofilm formation caused by the *L. nudicaulis* LE (Elkady et al., 2024) using the Equation 7.


(7) $$\mathrm{Biofilm change}\;\left(\%\right)=1-\frac{OD620\;\mathrm{of}\;NPs\;\mathrm{treated cells}\;}{OD620\;\mathrm{of}\;NPs\;\mathrm{untreated cells}}\;\times 100$$

### 2.8 Statistical analysis

The data obtained from all experiments, conducted in triplicate, were presented as the mean ± standard deviation (mean ± SD) and analyzed using SPSS for Windows version 20 (Statistical Package for Social Services, Chicago, IL, United States). The graphs were prepared using GraphPad Prism statistical software version 8. The activity index was determined to compare the antibacterial activity of the *L. nudicaulis* LE with standard antibacterials. In addition, the MIC index and therapeutic index were calculated to assess the antibiosis activity and biosafety of the *L. nudicaulis* LE, respectively.

## 3 Results and discussion

### 3.1 Confirmation and screening for carbapenem-resistant *P. aeruginosa*

Twenty-four *P. aeruginosa* clinical isolates, designated as P_1_–P_24_, were identified. These phenotypically confirmed non-lactose fermenting, non-hemolytic isolates were primarily characterized as motile, Gram-negative bacteria, with typical yellowish-blue pigmented, large, round, and opaque colonies with irregular margins and a distinctive fruity odor. In addition, these isolates were able to grow aerobically on cetrimide agar and MacConkey agar at either 40 or 42°C, as well as on TSI agar, producing a red slant and butt. Furthermore, phenotypic characterization were confirmed by the positive results for oxidase, catalase, and citratase enzyme production. According to the broth microdilution assay findings in our study, four isolates, P_8_, P_21_, P_22_, and P_23_, were categorized as carbapenem-resistant *P. aeruginosa*. Similarly, the *P. aeruginosa* clinical isolates were initially identified in the microbiology laboratory based on their motility and growth behavior on MacConkey and blood agar plates, and they were further identified according to their characteristic biochemical patterns in the oxidase, catalase, citrate, and TSI tests (Abdi et al., 2024). In addition, the *P. aeruginosa* isolates were selectively recovered using cetrimide agar. All isolates grew as a non-lactose fermenter on MacConkey agar and produced pigments on MHA, exhibiting a diagnostic yellowish-green to bluish-green color and a grape-like odor. Biochemically, these isolates were strongly positive for the production of oxidase, catalase, and citratase enzymes (Ezeador et al., 2020). These results underscored the importance of microscopic, cultural, and biochemical characteristics in the phenotypic identification of *P. aeruginosa* clinical isolates.

### 3.2 Phytochemicals screening of the *L. nudicaulis* LE

The results shown in Tables 1, 2 indicated the presence of compounds such as flavonoids, polyphenols, alkaloids, and quinones in LN. Furthermore, LN exhibited significant levels of various bioactive compounds such as flavonoids, phenolic acids, tannins, alkaloids, saponins, and steroids. Our findings align with those of Rani et al. (2023), who found that the methanol extract of LN contains a significant number of flavonoids. These compounds are often associated with antioxidant, anti-inflammatory, and other health-promoting properties (Hilal et al., 2024). The presence of glycosides, polyphenols, and other bioactive compounds suggests potential applications in pharmaceuticals, nutraceuticals, or functional foods. The results shown in Tables 1, 2 also indicated the absence of cardiac glycosides, anthraquinones, and anthocyanins. The phytochemicals in LN vary according to the type of solvents used: Aspidofractinin and tocopherol were detected in the chloroform extract of LN (Ramdan and Abdallah, 2017), while dihydro-isosteviol-methylester and flavone-4’-OH,5-OH,7-di-O-glucoside were found in the methanol extract of LN (Ramdan and Abdallah, 2017).

**Table 1:** Qualitative preliminary phytochemical screening of *L. nudicaulis.*

No	Test	<	No	Test	Results^*^
1	Flavonoids	++	9	Diterpenes	+
2	Cardiac glycosides	−	10	Quinones	++
3	Alkaloids	+	11	Tannins	+
4	Steroids	+	12	Phlobatannins	−
5	Saponins	+	13	Gums	−
6	Polyphenols	++	14	Anthocyanins	−
7	Anthraquinones	−	15	Carbohydrates	++
8	Glycosides	++	16	Proteins and amino acids	+

*+++, strong; ++, moderate; +, weak; –, completely absent.

**Table 2:** Quantitative phytochemical analysis of *L. nudicaulis.*

Item	Parameters	Results
1	Total flavonoids (mg RTE/g dry weight)	150.93 ± 0.78
2	Total flavonols (mg RTE/g dry weight)	85.93 ± 1.40
3	Total phenolic acids (mg GAE/g dry weight)	56.58 ± 0.80
4	Total tannins (mg GAE/g dry weight)	26.07 ± 0.44
5	Total alkaloids (mg/100 g dry weight)	0.53 ± 0.10
6	Total saponins (mg/100 g dry weight)	1.42 ± 0.12
7	Total steroids (mg/100 g dry weight)	0.728 ± 0.12

### 3.3 GC–MS of the *L. nudicaulis* LE

**Figure 1:**
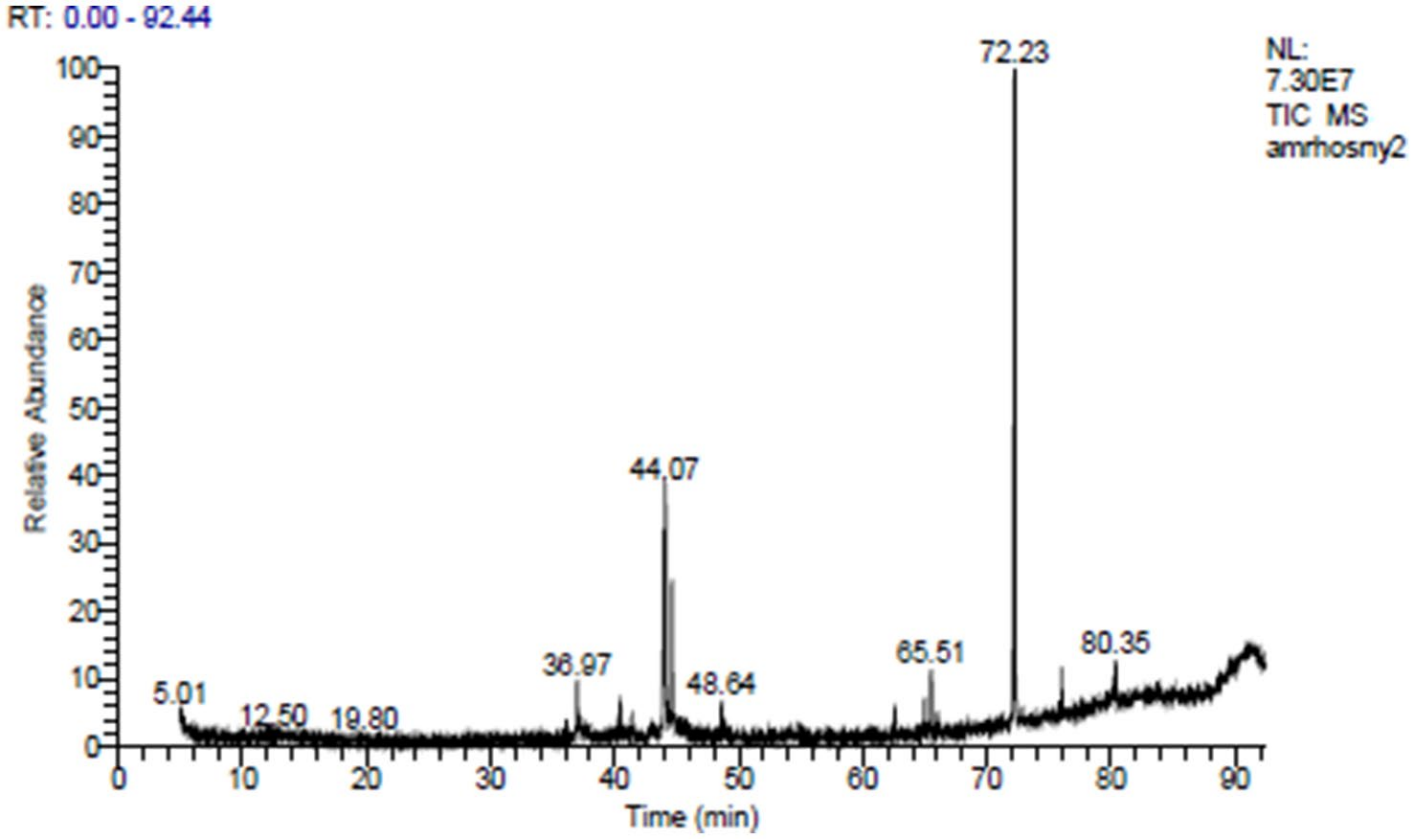
GC–MS of the *L. nudicaulis* LE.

The ethyl acetate extract of the LN plant contained 27 compounds that were identified by the GC mass analysis as being critically important. The major compounds included 7-acetyl-6-ethyl-1,1,4,4-tetramethyltetralin (20.61%), isopropyl myristate (7.69%), thiocarbamic acid, N,N-dimethyl, S-1,3-diphenyl-2-butenyl ester (6.48%), hahnfett (3.56%), cyclopentane acetic acid, 3-oxo-2-pentyl-, methyl ester (2.74%), hexadecanoic acid (2.58%), and dotriacontane (2.06%) (Table 3 and Figure 1). The compound 7-acetyl-6-ethyl-1,1,4,4-tetramethyltetralin, characterized by its tetralin molecular structure, is notable for its potent antioxidant and antimicrobial activities (Bester, 2009). Isopropyl myristate, a fatty acid ester, is widely employed in cosmetics and pharmaceuticals owing to its ability to moisturize and reduce friction through anti-inflammatory effects (Purnamawati et al., 2017). The thiocarbamic acid functional group consists of a sulfur atom double-bonded to a carbon atom, with two organic substituents attached. Thiocarbamic acid derivatives have been investigated for potential uses as pesticides, fungicides, pharmaceuticals, and in industrial applications (Ajiboye et al., 2022). A cyclopentane ring provides a rigid, cyclic scaffold that can influence the three-dimensional conformation and intermolecular interactions of a molecule. This can be important for binding to biological targets. The presence of the 3-oxo and 2-pentyl substituents on the cyclopentane ring further diversifies the chemical structure and enhances the potential biological activities, including anti-inflammatory, analgesic, antimicrobial, and anticancer effects, among others (Asif, 2016). A 16-carbon chain length is typical for many naturally occurring fatty acids, providing a good balance of physicochemical properties for biological functions. Hexadecanoic acid exhibits inhibitory effects against certain bacteria, fungi, and viruses (Orhan et al., 2011). The ethyl acetate extract of *Launaea nudicaulis* has been found to have several medicinal uses, including antidiabetic activity, pharmacological effects, and nutraceutical relevance (Mashlawi et al., 2024).

**Table 3:** GC–MS of the *L. nudicaulis* LE.

No.	Compound	RT (min)	Peak area %	Activity	References
1	1,3-Benzodioxole, 5-[1-[2-(2-butoxyeth oxy)ethoxy]butyl]	34.57	0.28	Insecticide	Ugolini et al. (2005)
2	Cyclopentane acetic acid, 3-oxo-2-pentyl-, methyl ester	36.12	0.84	Antimicrobial agent and plant defense mechanism	Chandrasekaran et al. (2008)
3	9-Octadecenoic acid (Z)-	36.34	0.34	Antioxidant and anticancer	El-Fayoumy et al. (2021)
4	Cyclopentane acetic acid, 3-oxo-2-pentyl-, methyl ester	36.98	2.74	Antimicrobial, antihypertensive, insecticidal, and antitussive	Sugden and Razdan (1972)
5	Methyl jasmonate	37.94	0.52	Anticancer, anti-inflammatory, and plant diseases suppression	Kitaoka et al. (2012)
6	2-Hexanone, (2,4-dinitrophenyl)hydrazone	38.23	0.16	Antimicrobial, antioxidant, and anti-inflammatory	Soukup et al. (1964)
7	Oleic acid	39.16	0.25	Antimicrobial, antioxidant, and apoptotic	Alabi et al. (2018), Hashem et al. (2023)
8	Octanal, 2-(phenylmethylene)-	40.48	1.42	Antimicrobial, anti-inflammatory, and antioxidant	Qiao et al. (2022)
9	Ambrosin	41.38	0.99	Alzheimer disease inhibition and minimizes inflammation and oxidative stress	Khalil et al. (2019)
10	Isopropyl myristate	43.96	7.69	Cosmetics, personal care, and biodegradability	Vadgama et al. (2015)
11	7-Acetyl-6-ethyl-1,1, 4,4-tetramethyltetralin	44.08	20.61	Solubilizer and emulsifier in pharmaceutical formulations and enhances the bioavailability of active pharmaceutical ingredients	Hillard Iii and Vollhardt (1977)
12	Quinindoline	44.86	1.14	Antimicrobial, anticancer, and antiviral	Mendez et al. (2018)
13	Mangostine	46.68	0.24	Antioxidant, anti-inflammatory, anticancer, antimicrobial, and Cardiometabolic benefits	Matsumoto et al. (2003), Shan et al. (2011), Sianglum et al. (2011), Vadgama et al. (2015)
14	Hexadecanoic acid	47.13	2.58	Antioxidant and antimicrobial	Sharaf et al. (2022)
15	Kauren-19-oic acid	47.63	0.18	Plant growth regulation and anti-inflammatory	Cavalcanti et al. (2009)
16	8-Octadecenoic acid	52.39	1.16	Cardiovascular health, anti-inflammatory, and skin health	Pacheco et al. (2008), Calder (2010), Jijie et al. (2017), Attia et al. (2023)
17	Isochiapin B	52.96	0.11	Anti-insect, antimicrobial, and antioxidant	Hashem et al. (2022), Quranayati et al. (2022), Nascimento et al. (2023)
18	Stearic acid	54.82	0.48	Anti-inflammatory and antimicrobial	Pan et al. (2010), Abdelaziz et al. (2023a)
19	9-Octadecenamide	60.51	0.36	Neuroprotective and anti-inflammatory	Huidobro-Toro and Harris (1996), Verme et al. (2005), Abdelaziz et al. (2023b)
20	Cyclohexane,1,3,5-triphenyl	62.58	1.39	Anticancer and antimicrobial	Javaid et al. (2021)
21	Thiocarbamic acid, N,N-dimethyl, S-1,3-diphenyl-2-butenyl ester	64.99	6.48	Pesticidal and fungicidal	Alhasan et al. (2019)
22	Hahnfett	68.88	3.56	Antibacterial	Lotfy et al. (2016)
23	13-Docosenamide	72.34	0.31	Anti-inflammatory, antioxidant, and anticancer	Tamilmani et al. (2018)
24	Ethyl iso-allocholate	74.67	1.62	Antimicrobial	Malathi et al. (2017)
25	Dotriacontane	76.02	2.06	Antioxidant	Ibrahim et al. (2017)
26	Vitamin E	80.21	0.80	Antioxidant and maintaining overall health	Brigelius-Flohé and Traber (1999)
27	ç-Sitosterol	83.75	1.19	Antimicrobial and antifungal	Vengadeshkumar and Sharmila (2020)

### 3.4 Cytotoxicity and anticancer effects

**Figure 2:**
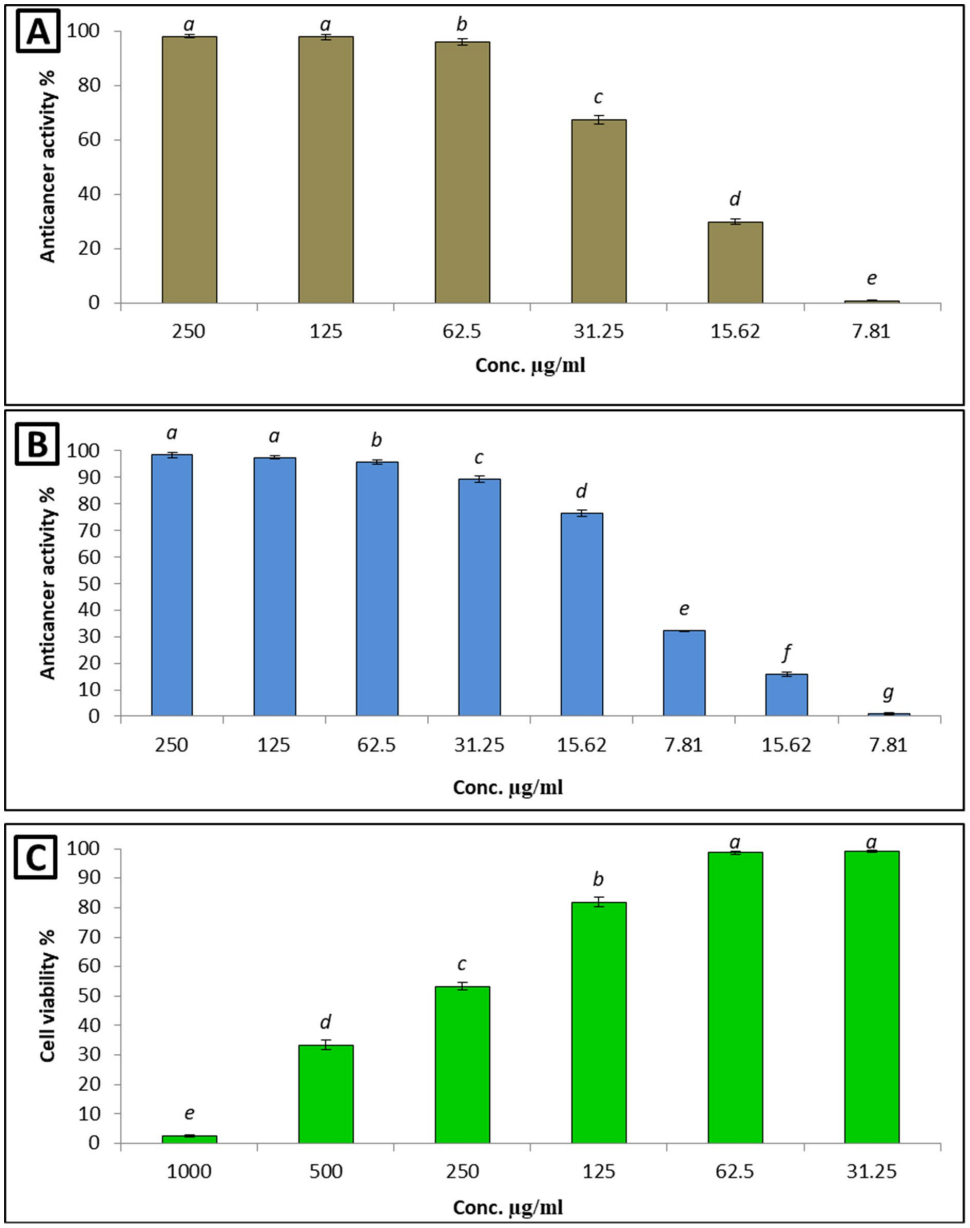
Anticancer effects of the different *L. nudicaulis* LE concentrations against Hep-G **(A)** and MCF-7 **(B)** cancerous cell lines, and its cytotoxicity on Wi-38 as a normal cell line **(C)**.

Chemotherapeutic agents that kill or inhibit the proliferation of cancerous cells have the drawback of being unable to differentiate between normal cells and malignant cells. As a result, they affect both cell types, leading to elevated toxicity and adverse effects (Zhong et al., 2021). In the present study, the low cytotoxicity of the *L. nudicaulis* LE, at different concentrations in the range of 31.25–1,000 μg/mL, toward the Wi-38 normal cell lines was evident. Notably, the viability of the cells improved with exposure to the elevated concentrations of the *L. nudicaulis* LE, with a promising inhibitory concentration 50 (IC50) value of about 262 μg/mL. Furthermore, the treatment of the MCF-7 and Hep-G2 malignant cell lines with the serially diluted *L. nudicaulis* LE in the range of 7.81–250 μg/mL demonstrated a significant anticancer activity. The viability percentage in both cell lines decreased after treatment with increased *L. nudicaulis* LE concentrations. Relatively low and promising IC50 values of approximately 12.7 and 24.5 μg/mL for MCF-7 and Hep-G2, respectively, were recorded (Figure 2).

Our findings align with those of (Mashlawi et al., 2024) a similar study conducted by Mashlawi et al. (2024), in which the *in vitro* cytotoxicity of the methanolic extract of *L. nudicaulis* against Wi-38, MCF-7, and HPG-2 cell lines was evaluated. Their results showed a concentration-dependent cytotoxic activity of the *L. nudicaulis* methanolic extract, with a relatively higher IC50 value of 46.30 and 38.56 μg/mL against MCF-7 and HPG-2 cell lines, respectively. However, their methanolic extract exhibited a simple inhibition (14.06%) on WI-38 normal cells. In addition, the ethyl acetate *L. nudicaulis* LE exhibited a respectable activity against another two cancerous cell lines, human colon carcinoma (HT-29) and human promyelocytic leukemia (HL-60), with IC50 values of approximately 10.7 and 12.40 μg/mL, respectively (Elhawary et al., 2023). These findings suggest that our *L. nudicaulis* LE had a specific ability to inhibit the proliferation of different cancerous cell lines with minimal harm to normal cells. This was likely due to the presence of several anticancer compounds, such as 9-octadecenoic acid, methyl jasmonate, quinindoline, mangostine, cyclohexane, 1,3,5-triphenyl, and 13-docosenamide (Table 3). Therefore, the *L. nudicaulis* LE could serve as a source of valuable, naturally derived, and efficient antitumor compounds.

### 3.5 Antibacterial activity

**Figure 3:**
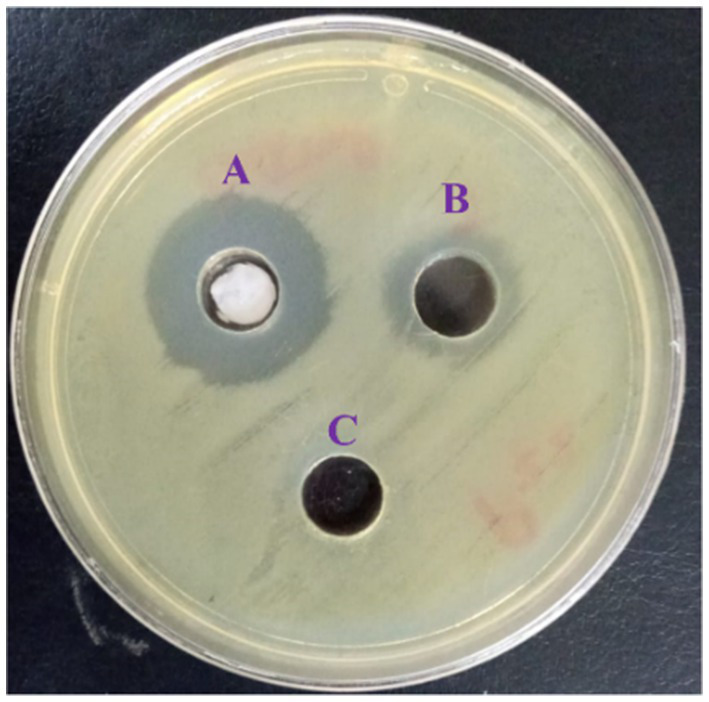
Agar well diffusion assay of the *L. nudicaulis* LE showing the *P.* zones of growth inhibition for the *aeruginosa* clinical isolate (P_22_). Wells **(A)** 30 μg/mL ceftriaxone (positive control), **(B)** 2000 μg/mL *L. nudicaulis* LE, and **(C)** DMSO (negative control).

The obtained behavior of the *L. nudicaulis* LE against the tested MDR *P. aeruginosa* clinical isolates from the well diffusion and broth microdilution assays is presented in Table 4. The initial antibacterial activity of the *L. nudicaulis* LE, represented in terms of the zone of inhibition, AI, and PI from the agar well diffusion assay findings (Figure 3), clarified the initial *in vitro* activity of the 2000 μg/mL ethyl acetate *L. nudicaulis* LE against 50% (12/24) of the *P. aeruginosa* clinical isolates. The susceptible isolates exhibited a mean IZD ranging from 11.34 ± 0.47 to 26.67 ± 0.47 mm. Interestingly, the obtained IZD of the ceftriaxone (30 μg/mL) positive control for these isolates was in the range of 15–29 mm. Accordingly, the calculated percentage inhibition of the *L. nudicaulis* LE against the susceptible isolates was in the range of 50–106.2%, indicating acceptable activity of our extract against the tested isolates. The highest antimicrobial activity, an IZD of 17 ± 0.82 mm and an activity index of 106.2%, with subsequent significant antibacterial effects was observed against the clinical isolate P_8_. Our results were in accordance with those of Khatri and Chhillar (2015) a relatively related study conducted by Khatri and Chhillar (2015). Their disc diffusion assay findings were dependent on the extraction solvent. Maximum IZDs of 14.95, 14.76, and 13.54 mm were recorded for a 25 mg/mL acetone extract against *Serratia marcescens*, *Staphylococcus aureus* (*S. aureus*), and *Salmonella enterica* ser. Typhi with PIs of 68, 59, and 74%, respectively. In another study, the evaluation of the antimicrobial activity of a 400 mg/mL methanolic extract of *L. nudicaulis* revealed an inhibitory effect in the form of variable IZDs of (6.5 ± 0.5 mm), (6.7 ± 0.3 mm), and (10.5 ± 0.5 mm) against *Escherichia coli* ATCC 35218, *S. aureus* ATCC 25923, and *S. epidermidis* ATCC 12228, respectively. In addition, when comparing these findings with the IZD of a standard gentamicin disc (10 μg), PIs of 29, 23, and 46% for *E. coli*, *S. aureus*, and *S. epidermidis*, respectively, were found (Ramdan and Abdallah, 2017). Furthermore, the maximum antibacterial activity of a 1 mg/well *L. nudicaulis* methanol extract was demonstrated in well diffusion assay against *Klebsiella pneumoniae*. The measured IZD was 22.5 mm for the methanol extract compared to an IZD of 25.5 mm for the positive control, 10 μg/well of ofloxacin, and the calculated PI was 88% (Rashid et al., 2000). Specifically, the agar diffusion assay clarified the antimicrobial activity of the essential oils from LN against *Klebsiella* sp., *Proteus* sp., *E. coli*, and *S. aureus* (Fadjkhi et al., 2020). The activity indices could be valuable for assessing the comparative antimicrobial effect of the tested plant extract and could help in deciding the importance of active constituent extraction (Assefa et al., 2024). Our PI findings highlight the promising effects of the evaluated LN extracts or their active constituents against Gram-negative bacteria. The activity of the *L. nudicaulis* LE against the *P. aeruginosa* clinical isolates could be correlated with the observed 7-acetyl-6-ethyl-1,1,4,4-tetramethyltetralin compound that possess potent antimicrobial activity and other active constituents shown in Table 3. In addition, the enhanced antibacterial activity of the *L. nudicaulis* extract could be mainly attributed to different biologically active phytochemical constituents, including terpenoids, flavonoids, tannins, alkaloids, glycosides, saponins, and anthraquinones, which vary depending on the extraction solvent. Synergistic interactions between these constituents could also contribute to the improved activity index of the tested extract. However, there is no evidence for the presence of a direct correlation between the anticancer and antibacterial activities of *L. nudicaulis* LE.

**Table 4:** Various anti-pseudomonas activity of the *L. nudicaulis* plant extract.

Character	*L. nudicaulis* LE	CRO	PI (%)	*L. nudicaulis* LE				
Isolates^*^								
	IZD (mm)			MIC	MBC	MIC index	Antibiosis effect	TI
				μg/mL				
P_1_	0	21	N/A	1,000	1,000	1	Bactericidal	0.262
P_2_	0	15	N/A	1,000	1,000	1		0.262
P_3_	26.67 ± 0.47	29	90.31	31.25	125	4	Bacteriostatic	8.384
P_4_	15.67 ± 0.47	25	64.0	15.625	125	8		16.768
P_5_	15 ± 0	26	57.6	1,000	1,000	1	Bactericidal	0.262
P_6_	24.34 ± 0.47	26	92.3	62.5	250	4	Bacteriostatic	4.192
P_7_	18.67 ± 0.47	23	82.6	62.5	500	8		4.192
P_8_	17 ± 0.82	16	106.2	62.5	250	4		4.192
P_9_	13 ± 0	25	52.0	125	500	4		2.096
P_10_	0	25	N/A	1,000	1,000	1	Bactericidal	0.262
P_11_	18.34 ± 0.47	24	75.0	62.5	500	8	Bacteriostatic	4.192
P_12_	0	0	N/A	1,000	1,000	1	Bactericidal	0.262
P_13_	11.34 ± 0.47	22	50.0	31.25	250	8	Bacteriostatic	8.384
P_14_	0	28	N/A	1,000	1,000	1	Bactericidal	0.262
P_15_	0	24	N/A	1,000	1,000	1		0.262
P_16_	0	31	N/A	1,000	1,000	1		0.262
P_17_	0	19	N/A	1,000	1,000	1		0.262
P_18_	0	25	N/A	1,000	1,000	1		0.262
P_19_	0	33	N/A	1,000	1,000	1		0.262
P_20_	0	25	N/A	1,000	1,000	1		0.262
P_21_	0	18	N/A	1,000	1,000	1		0.262
P_22_	11.67 ± 0.47	17	70.58	31.25	250	8	Bacteriostatic	8.384
P_23_	12.34 ± 0.47	15	80.0	62.5	250	4		4.192
P_24_	13.34 ± 0.47	22	59.09	31.25	250	8		8.384

*P_1_-P_24_, MDR *P. aeruginosa* clinical isolates (1–24); CRO, ceftriaxone; PI, percent inhibition; IZD, inhibition zone diameter; MIC, minimum inhibitory concentration; MBC, minimum bactericidal concentration; TI, therapeutic index; N/A, not applicable.

**Figure 4:**
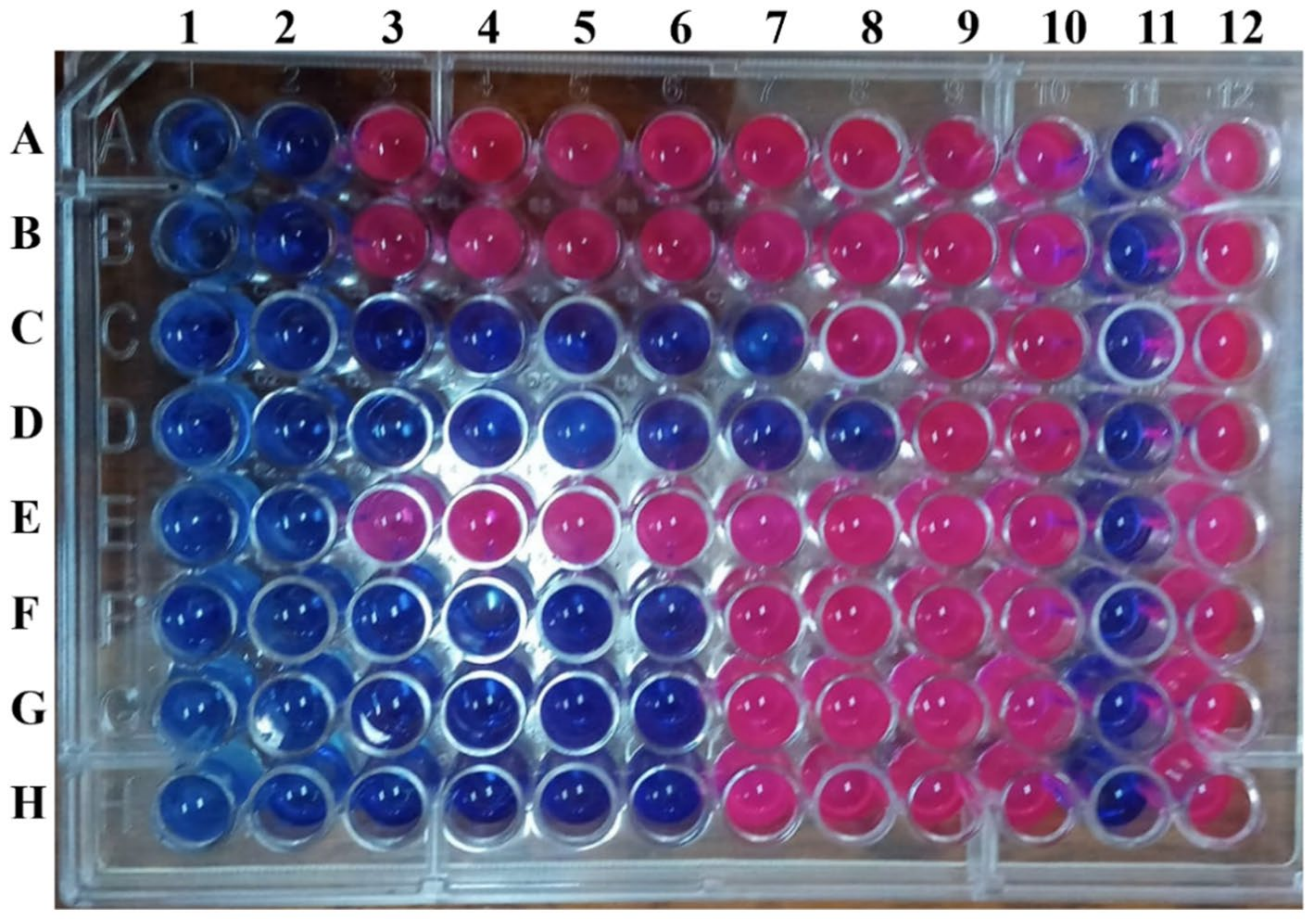
Resazurin-based broth microdilution assay illustrating the inhibitory activities of the *L. nudicaulis* LE. Rows A-D inoculated with *P. aeruginosa* P_1_-P_8_, respectively. Columns 1–10 containing the 2-fold serially diluted *L. nudicaulis* LE at the concentrations ranging from 2000 to 3.906 μg/mL. Columns 11 and 12 represent the negative and positive controls, respectively.

Quantitatively, the broth microdilution assay (Table 4 and Figure 4) showed the inhibitory activities of the *L. nudicaulis* LE against the MDR *P. aeruginosa* clinical isolates at different concentrations. The recorded low MIC values, ranging from 15.625 to 1000 μg/mL, demonstrated a high sensitivity of the tested isolates toward the *L. nudicaulis* LE. In addition, the tested isolates showed low MBC values, ranging from 125 to 1000 μg/mL, along with MIC indices ranging from 1 to 8. This indicated that both bacteriostatic and bactericidal antibiosis activities of the *L. nudicaulis* LE against the tested isolates depended on the concentration applied. At a high concentration (1000 g/mL), the *L. nudicaulis* LE exhibited bactericidal activity, while the bacteriostatic effect was observed at a lower concentration. Our findings align with (Khan et al., 2023) those of Khan et al. (2023), which reported the potent antibacterial activity of *L. nudicaulis* leaf, stem, and root methanolic extracts against *S. aureus*. In related research, methanol extracts of *L. nudicaulis,* rich in alkaloids, saponins, and flavonoids, exhibited antibacterial activity against *Streptococcus pneumoniae*, with an MIC value of 248 μg/mL (Nivas and Boominathan, 2015). In addition, the study conducted by Khatri and Chhillar (2015) reported high antibacterial activity of an LN leaves acetone extract against *Serratia marcescens*, *S. aureus*, and *Salmonella enterica* ser. Typhi, with MIC values of 190, 390, and 190 μg/mL, respectively, and MBC values of 190, 1560, and 190 μg/mL, respectively. The calculated MIC index of 1 for *Serratia marcescens* and *Salmonella enterica* ser. Typhi and 4 for of *S. aureus* indicated that the antibiosis mechanism of the extract was due to its bactericidal activity against the tested Gram-negative bacteria and its bacteriostatic activity against the tested Gram-positive bacteria.

Furthermore, the therapeutic indices of the tested *L. nudicaulis* LE calculated from its effect on a normal human cell line were in the range of 0.262–16.768. The higher therapeutic indices reflect its relative antibacterial selectivity toward the *P. aeruginosa* clinical isolates. In a relatively related study, the biosafety or low toxicity evaluation of newly tested antimicrobial agents was correlated with their recorded therapeutic indices, which ranged from 2 to 109 (Scaccaglia et al., 2024).

### 3.6 Antibiofilm activity

Regarding the biofilm formation side, the calculated ODc from the CV assay classified the tested 24 isolates into non (3/24; 12.5%), weak (2/24; 8.3%), moderate (11/24; 45.9%), or strong (8/24; 33.3%) biofilm-forming *P. aeruginosa* (Table 5). In addition, the majority (P_8_, P_22_, and P_23_) of the carbapenem-resistant isolates (3/4; 75%) were biofilm forming. In the research carried out by Lordelo et al. (2024), the tested *P. aeruginosa* strains were categorized according to their biofilm-forming capability into non-biofilm producers (3%), weak biofilm producers (9.1%), moderate biofilm producers (21.2%), and strong biofilm formers (66.7%). Another similar study reported a relatively comparable prevalence rate of 18.3, 18.3, 53.0, and 10.4% for strong, moderate, weak, and non-biofilm-forming *P. aeruginosa* isolates, and 95% of their tested carbapenem-resistant isolates were identified as biofilm formers (Heidari et al., 2022). The variation in biofilm-forming capacities among *P. aeruginosa* clinical isolates could be attributed to the types of collected specimens, the number of tested isolates, and the specific capabilities of each isolate for biofilm formation. Understanding the growth behavior and biofilm formation capacity of *P. aeruginosa* isolates could help in overcoming their possible medical device colonization and in ensuring proper eradication of this superbug.

**Table 5:** *P. aeruginosa* biofilm eradicative effect of the *L. nudicaulis* LE.

Item	Biofilm category	Biofilm percentage inhibition by the *L. nudicaulis* LE	Item	Biofilm category	Biofilm percentage inhibition by the *L. nudicaulis* LE
Isolates^*^			Isolates		
P_1_	Moderate	16.623 ± 0.62	P_13_	Moderate	24.222 ± 0.73
P_2_	Strong	17.721 ± 0.24	P_14_	Moderate	17.875 ± 0.86
P_3_	Weak	74.403 ± 0.42	P_15_	Moderate	12.653 ± 0.85
P_4_	None	N/A	P_16_	Strong	3.542 ± 0.07
P_5_	Strong	38.124 ± 0.61	P_17_	Moderate	4.224 ± 0.21
P_6_	Strong	51.170 ± 1.31	P_18_	Strong	11.647 ± 0.98
P_7_	None	N/A	P_19_	Moderate	17.625 ± 1.18
P_8_	Strong	30.452 ± 0.60	P_20_	Weak	4.089 ± 0.07
P_9_	Moderate	7.664 ± 0.44	P_21_	None	N/A
P_10_	Moderate	9.173 ± 0.79	P_22_	Moderate	50.892 ± 3.12
P_11_	Moderate	46.626 ± 0.4	P_23_	Strong	48.188 ± 1.2
P_12_	Strong	20.351 ± 0.75	P_24_	Moderate	47.541 ± 1.5

*P_1_-P_24_, MDR *P. aeruginosa* clinical isolates (1–24); N/A, not applicable.

Furthermore, this assay demonstrated the inhibitory activity of the *L. nudicaulis* LE against biofilm-forming *P. aeruginosa* in the range of 3.542 ± 0.07–74.403 ± 0.42%. In a related study, Alam et al. (2020) reported that extracts from Clematis grata and Bergenia ciliata, which are rich in catechin flavonoids, efficiently inhibited *P. aeruginosa* biofilm formation by 80 and 81%, respectively, without affecting viable growth. In addition, the catechin bioactive compound can reduce 72% of *P. aeruginosa* biofilms’ extracellular DNA content, an essential structural component of bacterial biofilm formation. Similarly, Song et al. (2018) found that negletein flavone, isolated from the leave extract of *Scutellaria oblonga*, exhibited a 72.3% *P. aeruginosa* biofilm-reducing ability at a concentration of 64 μg/mL. These findings underscore the potential application of plant extracts as antibacterial agents against biofilms, highlighting the role of bioactive constituents, such as flavonoids and other phenolic compounds, in minimizing bacterial adherence, which is the initial step in bacterial biofilm formation.

## 4 Conclusion

The development of various MDR bacterial species necessitates the development of potential antimicrobial alternatives. Accordingly, extensive research should be directed to uncover, isolate, and identify various bioactive constituents from widely distributed medicinal plants. The ethyl acetate extract of the *L. nudicaulis* LE was analyzed using GC–MS, identifying 27 key bioactive compounds, including 7-acetyl-6-ethyl-1,1,4,4-tetramethyltetralin, isopropyl myristate, and others. The LN was also found to be a rich source of various important phytochemicals, such as phenolic acids, tannins, saponins, and steroids. Our study reveals the anticancer activities of *L. nudicaulis* leaf extract, demonstrating low toxic outcomes in normal cells. On the microbiological side, the extract exhibited promising antibacterial activity, with high selectivity against *P. aeruginosa* clinical isolates, which could be help control antimicrobial resistance. The results also suggest that *L. nudicaulis* leaf extract is a promising candidate for future studies that plan to separate, purify, and identify its various active constituents and that plan to independently evaluate its possible therapeutic bioactivity for selecting the most potent therapeutic constituent. In addition, the findings highlight the need for further investigations to clarify the microbial targets and identify the destructive mechanism of the most active constituents.

## Data Availability

The original contributions presented in the study are included in the article/supplementary material, further inquiries can be directed to the corresponding authors.
